# Metabolization and sequestration of plant specialized metabolites in insect herbivores: Current and emerging approaches

**DOI:** 10.3389/fphys.2022.1001032

**Published:** 2022-09-27

**Authors:** Adriana Moriguchi Jeckel, Franziska Beran, Tobias Züst, Gordon Younkin, Georg Petschenka, Prayan Pokharel, Domenic Dreisbach, Stephanie Christine Ganal-Vonarburg, Christelle Aurélie Maud Robert

**Affiliations:** ^1^ Laboratory of Chemical Ecology, Institute of Plant Sciences, University of Bern, Bern, Switzerland; ^2^ Department of Insect Symbiosis, Max Planck Institute for Chemical Ecology, Jena, Germany; ^3^ Department of Systematic and Evolutionary Botany, University of Zürich, Zürich, Switzerland; ^4^ Boyce Thompson Institute, Ithaca, NY, United States; ^5^ Plant Biology Section, School of Integrative Plant Science, Cornell University, Ithaca, NY, United States; ^6^ Department of Applied Entomology, Institute of Phytomedicine, University of Hohenheim, Stuttgart, Germany; ^7^ Institute for Inorganic and Analytical Chemistry, Justus Liebig University Giessen, Giessen, Germany; ^8^ Department of Visceral Surgery and Medicine, Bern University Hospital, University of Bern, Bern, Switzerland; ^9^ Department for BioMedical Research, Visceral Surgery and Medicine, University of Bern, Bern, Switzerland

**Keywords:** plant specialized metabolites, detoxification, sequestration, -omics, resistance, tolerance, herbivory

## Abstract

Herbivorous insects encounter diverse plant specialized metabolites (PSMs) in their diet, that have deterrent, anti-nutritional, or toxic properties. Understanding how they cope with PSMs is crucial to understand their biology, population dynamics, and evolution. This review summarizes current and emerging cutting-edge methods that can be used to characterize the metabolic fate of PSMs, from ingestion to excretion or sequestration. It further emphasizes a workflow that enables not only to study PSM metabolism at different scales, but also to tackle and validate the genetic and biochemical mechanisms involved in PSM resistance by herbivores. This review thus aims at facilitating research on PSM-mediated plant-herbivore interactions.

## Introduction

The ability of insect herbivores to cope with plant specialized metabolites (PSMs) is an important driver of ecosystem functioning (Erb and Robert, 2016; Heckel, 2018; Beran and Petschenka, 2022). In particular, PSM detoxification and sequestration exert strong selective pressures on both lower and higher trophic levels (Erb and Robert, 2016; Beran and Petschenka, 2022). Understanding the mechanisms enabling herbivores to tolerate and sometimes even highjack plant defenses will shed light on natural food-web dynamics, and provide new avenues for pest management in agriculture. Yet, while the (agro)ecological impacts of PSM detoxification and sequestration by herbivores are well recognized, the involved biochemical mechanisms remain poorly understood.

Many PSMs are biologically active organic compounds that serve a primarily defensive function for the plant (but see Erb and Kliebenstein, 2020 for other roles of PSMs in plants). Upon tissue disruption, PSMs are often converted to more reactive forms under the action of plant and/or herbivore enzymes (Halkier and Gershenzon, 2006; Morant et al., 2008; Dobler et al., 2011; Pentzold et al., 2014; Erb and Reymond, 2019). Additionally, plants perceive the attack by detecting herbivore- and damage-associated molecular patterns (HAMPs and DAMPs), which trigger a cascade of signaling events, including membrane depolarization (Vm), increase of cytosolic Ca^2+^, production of reactive oxygen species (ROS), and activity of mitogen-activated protein kinase (MAPK) (for review see: Erb and Reymond, 2019). Perception and early signaling elicit the biosynthesis of phytohormones, such as jasmonic acid isoleucine (JA-Ile), that further regulate the production of PSMs (Erb and Reymond, 2019). PSMs and their activated products interfere with numerous targets in the herbivore body, resulting in effects ranging from anti-nutritional to toxic reactions (for review see Wink and Schimmer, 2010; War et al., 2019). For example, cardenolide defense compounds from plants of the milkweed family, specifically inhibit Na^+^/K^+^-ATPase, an essential ion carrier in animals, whereas isothiocyanates, the hydrolysis products of glucosinolates in brassicaceous plants, are broadly reactive towards biological nucleophiles (Brown and Hampton, 2011; Agrawal et al., 2012).

Herbivorous insects have evolved a plethora of strategies to withstand deleterious effects of PSMs (Pentzold et al., 2014; Heckel, 2018). These strategies can be classified as tolerance and resistance mechanisms. Tolerance mechanisms comprise adaptations that prevent damage by intact PSMs without any modification nor transport of the compounds, and include inhibition of PSM activation enzymes, insensitivity, exclusion, and compensation mechanisms. The inhibition of PSM activation can occur through the inhibition of the plant and/or insect β-glucosidases (activation enzymes). For example, it was suggested that the high alkaline midgut lumen (pH 11) of the fall webworm *Hyphantria cunea* prevents the hydrolysis of cyanogenic glucosides in the gut and protects the herbivore from adverse effects (Fitzgerald, 2008). Additionally, some insects, such as the yellow woolly bear and tiger moth caterpillars, reduce the activity of endogenous β-glucosidases when feeding on increasing amounts of the iridoid glucoside aucubin (Pankoke et al., 2010). Insensitivity refers to mechanisms that prevent PSMs from binding to their targets by altering the target binding site. The most famous example of insensitivity was described in the monarch butterfly, *Danaus plexippus*, which has a reduced target-site sensitivity to cardenolides due to two key mutations in its Na^+^/K^+^-ATPase (Aardema et al., 2012; Petschenka et al., 2013a). By preventing the inhibition of Na^+^/K^+^-ATPase, and thereafter the associated disorders in muscle contraction, neural function, and ion transport (Schatzmann, 1965; Aardema et al., 2012), the insect can tolerate cardenolides. Exclusion mechanisms include mechanisms that constrain PSMs to the digestive system until excretion, as well as PSM barriers formed around other tissues that contain PSM target sites. For example, the guts of the locust *Schistocerca gregaria* and of the cockroach *Periplaneta americana* are impermeable to both polar and nonpolar cardiac glycosides (Scudder and Meredith, 1982). In contrast, the oleander hawk-moth, *Daphnis nerii*, uses its perineurium surrounding the nerve cord to shield its nervous system from cardenolides, while the PSMs may be present in its hemolymph (Petschenka et al., 2013b). Finally, compensation is a strategy that alleviates PSM-mediated inhibition of insect enzymes through the over-expression of PSM targets.

On the other hand, resistance mechanisms involve the biotransformation of PSMs and/or active transport. Resistance yields reduced toxicity, diversion, or rapid elimination of the molecules. Resistance strategies include PSM metabolization, sequestration, and excretion of metabolized products. Metabolization entails the molecular conversion of PSMs in other, often less reactive, compounds. While the term “detoxification” is commonly used to refer to PSM metabolization in herbivorous insects, it implies that the metabolization product is less toxic to the herbivore than the intact one, a conclusion which is too rarely tested.

Metabolization processes are commonly divided in phase I, phase II, and phase III reactions (Meyer, 1996). Phase I involves oxidation, reduction, or hydrolysis of the PSMs into water-soluble, more polar, metabolites. P450 cytochromes, a family of membrane bound enzymes, were frequently found to be involved in PSM metabolization in insect herbivores (Nauen et al., 2022). Phase II corresponds to the addition of hydrophilic groups through methylation, glucosylation, acetylation, sulfation, or conjugation with amino acids or glutathione. Glutathione-S-transferases are multifunctional enzymes which catalyze the addition of the thiol group from the reduced glutathione to PSMs, resulting in more water-soluble compounds (Ketterman et al., 2011). Phase III refers to the transport of the metabolite. Representative superfamilies involved in phase III of biotransformation include ATP-binding cassettes (ABC) and solute carrier (SLC) transporters (Wu et al., 2019; Hilliou et al., 2021). PSMs and their metabolization products can further be sequestered or excreted by the herbivore. Sequestration requires the active transport of PSMs from the gut to other compartments. The transport of PSMs occurs rapidly and selectively after PSM ingestion, as PSMs such as some glucosinolates were observed to be taken up from the front gut part into the hemolymph of flea beetle *Phyllotreta armoraciae* and sawfly larvae *Athalia rosae* (Abdalsamee et al., 2014; Yang et al., 2022). Sequestration of intact PSMs has been suggested to be a strategy to circumvent the enzymatic activation of the compound in the gut. For example, sawfly larvae have an increasing myrosinase activity through the gut, and active removal of glucosinolates from the gut early on during digestion may limit their activation (Abdalsamee et al., 2014). Similarly, senecionine *N*-oxide is normally reduced to the more toxic senecionine under conditions found in the insect gut, but rapid sequestration of the *N*-oxide form by the flea beetle *Longitarsus jacobeae* can prevent its activation (Narberhaus et al., 2004). PSMs can be sequestered in different tissues of the insects, such as in the haemolymph, integument, fat body, or glands (Nishida, 2002; Abdalsamee et al., 2014; Robert et al., 2017; Li L et al., 2019). While the sequestration of PSMs can be considered as a resistance strategy, it must be coupled to additional tolerance strategies to prevent autotoxicity during transport and storage (e.g., absence or insensitivity of targets in the sequestering tissue). While the role of sequestration in insect PSM resistance remains under debate, its impact as protection of the insect against higher trophic levels was clearly demonstrated in several models (Opitz and Müller, 2009; Erb and Robert, 2016; Beran and Petschenka, 2022). Finally, rapid excretion of PSM metabolization products contributes to lower PSM toxicity. The red palm weevil *Rynchophorus ferrugineus* and the aphid *Myzus persicae* conjugate electrophilic PSM molecules with reduced glutathione (GSH), thereby increasing their solubility and excretion rate (Francis et al., 2005; AlJabr et al., 2017). Noteworthy, excretion may occur via water-soluble as well as volatile exudates (Kumar et al., 2014; Robert et al., 2017). All herbivore mechanisms to tolerate and/or resist PSMs are not mutually exclusive and usually occur in an intricate manner in specialized herbivores.

This review focuses on insect resistance to PSMs and provides a guide of current and emerging technologies that can be employed to characterize PSM metabolism, sequestration, and active excretion in insects. In particular, this review highlights how to depict PSM Absorption, Distribution, Metabolism, and Excretion (ADME profile (Doogue and Polasek, 2013; Zhang et al., 2022), Figure 1) from sample preparation to mechanism validation. Finally, it identifies methods developed in other fields (*e.g.* medicine and machine learning) that could be applied to plant-herbivore interactions and increase our knowledge about PSM detoxification and sequestration.

**FIGURE 1:**
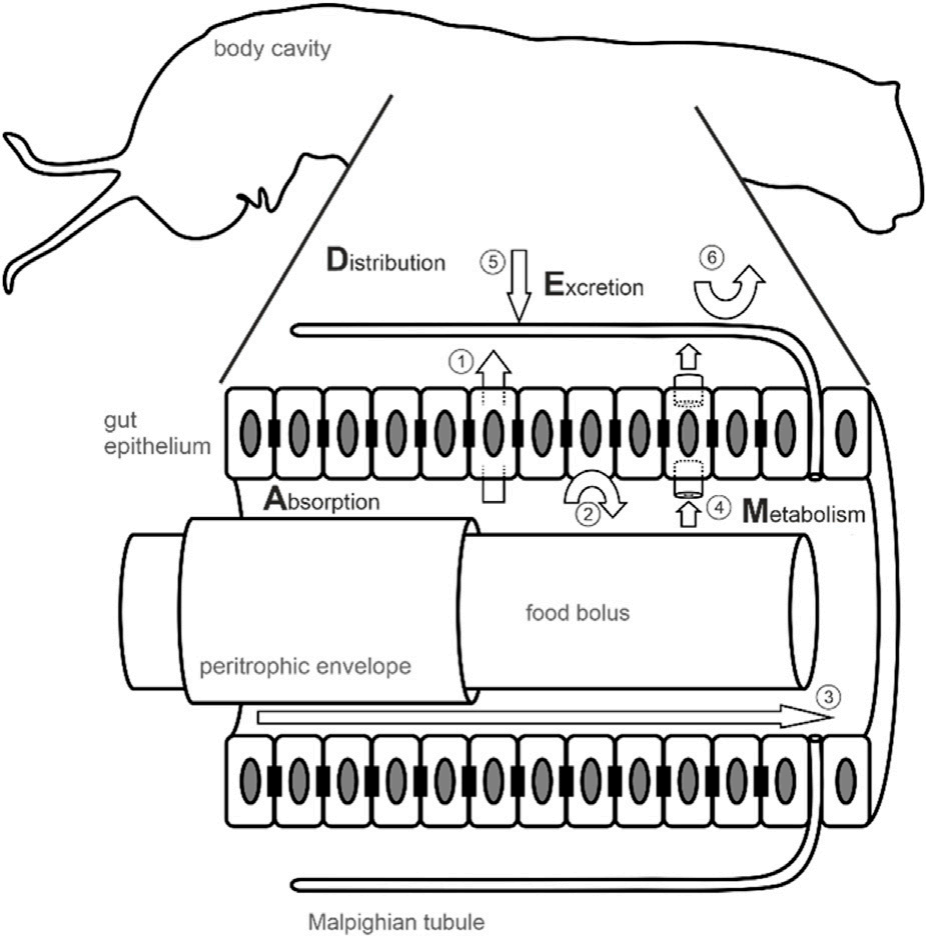
Metabolic fate of PSM in the insect body after oral uptake. How ingested PSM will behave in an insect body is characterized by the absorption of PSM from the gut lumen into the hemocoel, its distribution across insect body tissues, metabolism (i.e. enzymatic alteration), and excretion and is summarized by the acronym ADME. The figure shows a schematic insect body (caterpillar of the monarch butterfly) with an enlarged and detailed illustration of the digestive tract. In many insects, the food bolus is ensheathed by a peritrophic envelope which is secreted by the enterocytes of the gut epithelium. Besides protecting the gut epithelium against abrasion, the peritrophic envelope represents a first barrier preventing aggregates of nonpolar PSM to cross the gut epithelium (Barbehenn, 1999). Within the gut lumen, but also in the hemocoel (e.g. in the fat body which is not shown or in the hemolymph) PSM can be metabolically altered by enzymes. The insect gut epithelium is a monolayer of cells (enterocytes, shown as rectangles with an elliptical nucleus) mediating the uptake of nutrients and PSM in sequestering insects. In addition, it forms a barrier preventing the uptake of PSM by septate junctions (black rectangles) connecting the enterocytes and maintaining a diffusion barrier supposedly blocking the paracellular route for PSM. Nonpolar PSM likely can cross the gut epithelium passively by diffusion (1). Active barriers (2) presumably mediated by efflux carriers such as multidrug resistance proteins (Dobler et al., 2015) are predicted to prevent nonpolar PSM from crossing the epithelium and protect target sites located in the hemocoel. Taken together, septate junctions and efflux carriers are predicted to render the gut epithelium impermeable to PSM leading to excretion of unaltered PSM via defecation (3). In sequestering insects, PSM may be transported by carrier proteins located in the gut epithelium (4). After functionalization (phase I reactions) and conjugation (phase II reactions), PSM are excreted via the Malpighian tubules (5). Reabsorption of PSM from the Malpighian tubules into the hemocoel (6) prevents clearance and supports retention of PSM in sequestering insects (Yang et al., 2021).

## Sample preparation

### Insect diet

Elucidating the mechanisms underlying detoxification and/or sequestration in insects often requires manipulation of PSM levels in the herbivore diet. Varying levels of PSMs can be obtained by complementing artificial diets, painting or spiking of leaves/stems/roots, or using mutant plants.

Artificial diets are an excellent tool to control insect nutrition both qualitatively and quantitatively. Artificial diets are typically agar-based and contain plant material such as wheat germ, bean powder, pulverized dried foliage, or purified individual components at defined concentrations (*e.g*., liquid diets for aphids), resulting in diets of different complexity. While diets based on host plant material may match the insects’ dietary requirements most closely and likely contain important chemical feeding stimuli, they will also contain PSMs and/or enzymes that metabolize PSMs. Using purified diet, *e.g*., produced from essential and controlled ingredients only, avoids the presence of PSMs and ‘undesirable’ degrading enzymes and reduces the diet batch-to-batch variability. On the other hand, artificial diet composition can also affect activity of insect digestive enzymes. For example, the activity of glucose oxidase is ten times higher in caterpillars of the beet armyworm, *Spodoptera exigua,* when raised on a wheat germ-based artificial diet compared to when raised on a host plant (Merkx-Jacques and Bede, 2005). After selecting an appropriate diet, the process of PSM complementation should be carefully considered. In particular, the stability of the PSMs will determine whether complementation can be performed during diet preparation (*e.g.*, if the PSMs are stable at elevated temperature). Limited solubility of many lipophilic PSM in water-based diets may require dissolving PSM in organic solvents which need to be included into PSM-free control diets as well to correct for toxic effects of the solvent (Pokharel et al., 2021). If PSMs are observed to degrade during diet preparation, application of PSMs to the diet surface prior to feeding assays may be an alternative solution. In any case, the stability of the compound in or on the surface of diets should be verified. An elegant strategy to control the delivery of very unstable PSMs is to complement the diet with a stable precursor and to add activating enzymes at regular intervals (Maag et al., 2014). While the importance of choosing the appropriate artificial diet recipes and complementation methods is established in mammal models (Pellizzon and Ricci, 2018), further efforts to establish adequate insect diets should be pursued.

If artificial diets are not available for an insect system, leaf disks from accepted host plants can be painted or spiked with a PSM. For example, caterpillars of the monarch butterfly can be fed cardenolide compounds on discs of the cardenolide-free milkweed plant Asc*lepias tuberosa* (Agrawal et al., 2021), while the mustard beetle *Phaedon cochelariae* can be fed glucosinolates on leaf discs of pea (Friedrichs et al., 2022). The PSMs are painted onto leaf discs dissolved in an organic solvent which evaporates, leaving only the PSM behind. Nonetheless, control leaf discs should be painted with PSM-free solvent to account for any potential solvent-related changes in the leaf disc.

For an increasing number of plant systems, mutants can be obtained by biotechnological tools in which PSM production is silenced, overexpressed, or even introduced *de novo*. Current methods for mutagenesis include chemical mutagenesis (*e.g.*, ethyl methanesulfonate), radiation mutagenesis (X-rays, fast neutrons, beta irradiation and UV), insertional mutagenesis (transposable elements, transferDNA, targeted induced local lesions in genomes (TILLING)), RNA interference (RNAi), and Clustered Regularly Interspaced Palindromic Repeats-CRISPR-associated protein 9-based (CRISPR/Cas9) genome editing technology. An elegant review on the latest progress in plant transformation was recently published (Kausch et al., 2019).

Existing mutant lines can be identified and obtained from germplasm libraries. For example, *Arabidopsis* mutants can be found at the *Arabidopsis* Information Resource (TAIR (Berardini et al., 2015)), the RIKEN *Arabidopsis* Genome Encyclopedia II (RARGE II (Akiyama et al., 2013)), or the Arabidopsis Acyl-lipid Metabolism Pathway database (ARALIP (McGlew et al., 2015)). Maize germplasms of ethyl methanesulfonate (EMS) Inflorescence Project Mutants and of Sequence-tagged insertion mutants (UniformMu, RescueMU, Ac/Ds/Ds-GFP) are available at the Maize Genetics and Genomics database (Woodhouse et al., 2021). Mutants of *Brachypodium*, an emerging plant model, are available at the Joint Genome Institute (T-DNA, chemical, and radiation mutants (Fitzgerald et al., 2015; Raissig and Woods, 2022). Additional mutant libraries were further produced for numerous plants, including tobacco (Julio et al., 2008; Lewis et al., 2010, 2015; Takakura et al., 2018; Udagawa et al., 2020; Zhao et al., 2020), tomato (Saito et al., 2011), rice (Meng et al., 2017), or cotton (Lian et al., 2020).

Finally, near isogenic lines (NILs) may also be created if naturally occurring PSM mutants are available (Muehlbauer et al., 1988; Young et al., 1988). PSM mutation can be introgressed from a donor parent (natural mutant) into a recurrent line. The introgression is performed by backcrossing individuals carrying the mutation back into the recurrent line over six to seven plant generations. The presence of the mutation is then stabilized by selfing the generated seedlings and identifying homozygotes at the target locus. This process leads to the production of a mutant NIL that is mostly identical to the wild type, except at the region surrounding the gene of interest. NILs can easily be produced in species with small genomes and short generation times, and their use is particularly relevant for gene mapping and function analysis.

Manipulating PSM exposure of insects via plants diet comes with some limitations that should be taken into account. For example, PSMs may occur *in planta* within specific storage structures, thus spiking of PSMs into artificial diets or onto leaf discs may not result in normal activity. Furthermore, molecular manipulation of host plant PSM profiles may have pleiotropic effects, either directly due to the transformation procedure, or due to the multiple roles of PSMs *in planta*. It has been recognized that some PSMs are involved in primary plant processes such as signaling, nutrition, and growth (Erb and Kliebenstein, 2020). Altering the production of some PSMs may thus yield changes in the plant’s primary metabolism, and thus in plant quality for an herbivore. For example, the benzoxazinoid 2,4-dihydroxy-7-methoxy-(2H)-1,4-benzoxazin-3(4H)-one (DIMBOA) is exuded by maize roots in the rhizosphere, where it acts as a siderophore chelating iron (Fe) (Hu et al., 2018). The resulting Fe-DIMBOA complexes are used for plant nutrition and are crucial for Fe homeostasis (Hu et al., 2018). A mutation in the DIMBOA biosynthesis pathway was reported to be associated with leaf chlorosis and decreased plant growth, and eventually, reducing the plants’ nutritional quality for a herbivore (Hu et al., 2018). Pleiotropic effects should be carefully assessed and addressed during any assays using mutant plants.

### Experimental design

Feeding assays to characterize detoxification and sequestration should be carried out in a standardized fashion. Concentrations of PSMs in insect bodies may not relate linearly to body size or time of PSM exposure, and can additionally vary substantially across larval instars (Jones et al., 2019). Consequently, similar sized insects of the same instar should be used for all treatment comparisons, and time of exposure to PSMs should be kept constant within and across treatments. In insects such as caterpillars, larval instars can be identified by the size of the head capsule (George and Hintz, 1966; Calvo and Molina, 2008; Castañeda-Vildózola et al., 2016; Sukovata, 2019). Exposure to PSMs can be approximated by intake of diet (accounting for loss by evaporation), consumption of plant material, or alternatively by the amounts of excretion products (*i.e*., fecal stains of bugs on filter paper, or mass of caterpillar frass, see Section 2.3). Finally, sequestration and detoxification of PSMs should be studied in actively feeding insects which are not undergoing molting or diapause.

For studying the kinetics of sequestration or as a control for predator-prey studies, it is commonly a prerequisite to generate PSM-free insects. While this can be achieved by using PSM-free artificial diets or mutant plants devoid of PSMs (See Section 2.1), creating PSM-free specimens will remain challenging, especially for specialized insects feeding on non-model host plant species, and for species capable of maternal transfer of PSMs, as these will require more than one generation of PSM-free rearing. Alternative approaches may involve selection of lineages on different, PSM free, host species. For instance, Brower et al. (1967) produced cardenolide free monarch butterflies raised on cabbage.

Testing for sequestration in intact insect specimens (*i.e.*, without dissecting) requires avoiding contamination by gut contents which otherwise could result in false positive results. Starvation of insects prior to analyses should reduce the amount of plant chemicals in the insect gut but may be an insufficient approach given that insects can accumulate and retain plant toxins in the gut [as recently described for cardenolides in monarch butterfly caterpillars (Dreisbach et al., 2022)]. Alternatively, insects may be fed a PSM-free diet to more actively purge their gut from remaining PSMs. However, short term starvation or purging might be insufficient: extracts from non-sequestering milkweed bug species fed with radioactively labeled (^3^H) cardenolides, followed by feeding on toxin-free sunflower seeds for 3 days still contained the radioactive label, which was substantially reduced after 10 days of feeding on sunflower seeds (Bramer et al., 2015). For these reasons, it is advisable to remove guts from insect bodies by dissection before extraction for chemical analyses.

### Collecting insect organs, fluids, and frass

Following the fate of PSMs after herbivore ingestion requires collecting insect tissues, body fluids such as saliva and hemolymph, and excretions for chemical analyses.

Collecting insect frass in a standardized manner is a challenging task. For some insects, such as *Manduca* or *Spodoptera* caterpillars, large pellets can be directly collected with forceps and using dropping frass traps (Zandt, 1994). Yet, the dehydration of the pellet following excretion should be standardized by using dry masses for analyses. In addition, the time between defecation and collection should be minimized to avoid breakdown of labile compounds by insect, plant, or microbial enzymes or by exposure to ambient conditions. The challenge of collecting frass from small insects can be overcome by placing several individuals together in/on a delimited surface that can further be used for PSM extraction. For instance, one can place the insects in Eppendorf tubes or on a filter paper for the collection period and extract the PSMs from these surfaces (Robert et al., 2017). Besides screening for potential metabolites, this method can also be used for qualitative verification of feeding activity on a toxic diet, *e.g*. when it is unclear if a diet spiked with PSM was actually consumed by the insects.

Dissecting insects should be preferentially carried out using alive or freshly killed specimens to prevent dislocation of analytes across body compartments due to tissue disruption (*e.g.* of the gut epithelium) by ice crystals during freezing. For dissecting, insects should be anesthetized by chilling on ice which may work differentially well for different insect species. For example, caterpillars from warmer regions such as the monarch butterfly will remain motionless for a longer period of time compared to caterpillars from temperate regions (as observed for selected species of geometrid caterpillars). Using a chilled dissection stage such as Petri dishes filled with crushed ice would be a solution to overcome this problem. As an alternative to chilling, insects can be anaesthetized with CO_2_. During dissection, care needs to be taken not to puncture the intestine to prevent plant compounds from spilling over into other body compartments. Therefore, it is advisable to leave the head and anus attached to the gut before taking it out to remove leakage of PSM containing gut contents into other body tissues whenever possible. Lepidopteran caterpillars frozen and stored at −80°C in tightly sealed containers that are dissected immediately after thawing display very well-preserved tissue morphology, so that dissection of insects after storing might represent a valid alternative to dissecting live specimens. For chemical analyses of diffusible PSM within tissues, it may be advisable to dissect specimens without immersion in a buffer to prevent a washout of analytes. Depending on the research questions to be addressed, especially organs involved in detoxification such as the fat body or the Malpighian tubules might be of special interest. For the analyses of macromolecules such as enzymes or RNA, dissecting under a physiological buffer such as PBS (phosphate buffered saline) will greatly facilitate the recovery of fragile organ systems. Besides tissue disruption, freezing and thawing will also increase degradation of labile plant compounds, especially secondary active glucosides easily hydrolyzed by enzymes. One recent approach to overcome both analyte dislocation and degradation is to dissect insect specimens after freeze drying. This method has been used successfully for dananine caterpillars (Petschenka and Agrawal, 2015) but might be useful for a range of insects. To study the fate of plant compounds during the insect gut passage in caterpillars (and probably other insect larvae or adults), dissecting freeze-dried insect specimens should be replaced by a modified approach based on freeze drying guts dissected from fresh caterpillars (Dreisbach et al., 2022). During this procedure, live caterpillars are dissected under ice-cold PBS to expose the gut. Importantly, the entire preparation remains constantly immersed to prevent collapsing of the gut and dislocation of compounds. After washing, the entire preparation including the buffer will be frozen and freeze dried. Finally, precipitated salts can be brushed off and guts can be subdivided for chemical analysis.

## PSM metabolic fate in insects

### Targeted metabolomic analyses

Targeted metabolomics can be used to determine the fate of PSMs as they are consumed, stored, or excreted by the insect. In addition, where pathways of PSM modification or degradation in insects are known, targeted metabolomics also allows to track and quantify these known metabolization products (*e.g.*, deglucosylated metabolites). Targeted metabolomics thus is a useful tool for PSM budget analyses and can provide information on the efficiency of detoxification/sequestration, and even on the use and efficiency of specific metabolic pathways by different insect species. If major discrepancies appear between PSMs and their known metabolic products in such an analysis, an untargeted metabolomic approach may become necessary, ideally combined with feeding assays using labeled compounds (see Section 3.2 and Section 3.3, respectively, for more information). For example, Jeschke et al. (2017) compared the metabolic fate of labeled glucosinolates in specialized and generalist herbivores. The generalist herbivores excreted most of the metabolites as the expected activated isothiocyanate compounds. However, 7–25% of excreted products were unknown compounds that would have been missed by targeted analyses. In contrast, two specialized herbivores detoxified approximately 90% of the glucosinolates to either desulfo-glucosinolates and/or nitrile, two non-toxic compounds. This comparison thus further shows that different herbivore species use distinct pathways for detoxification, and that even specialized species may employ different strategies to cope with PSMs.

As the aim of targeted metabolomics is to detect a specific group of compounds, the choice of extraction and analysis methods can be optimized to prioritize detection of these target metabolites. In fact, for some compounds such as glucosinolates, protocols for compound detection by High-Performance Liquid Chromatography and UV detection (HPLC-UV) have been well-established (Grosser and van Dam, 2017), and are commonly used for detection of glucosinolates in insect bodies or excretion products (*e.g.*, Kim and Jander, 2007). For most other metabolites, mass spectrometry (MS) is a key detection method, due to the high sensitivity of MS instruments allowing for the detection of compounds at very low concentrations, often with little sample preparation and concentration requirements. MS-based targeted metabolomics relies on previously elaborated databases, with information on the PSM of interest’s chemical formulae, retention times, and exact masses of parent ions and all major compound fragments or “daughter” ions (Fiehn, 2002). MS detection is commonly coupled with chromatographic separation of compounds prior to analysis in liquid (LC-MS) or gas phase (GC-MS) as an important step to separate and simplify the often highly complex mixtures of compounds in biological samples, and to thereby facilitate interpretation of the resulting mass spectrometry data. Separation also avoids potential issues of signal suppression by co-detected masses (“signal quenching”).

Volatile and non-thermosensitive compounds are best analyzed by GC-MS, either by directly adsorbing volatiles to a matrix before thermal desorption (*e.g.*, isothiocyanates, Friedrichs et al., 2022), or by sample extraction in a volatile solvent such as hexane (*e.g.*, alkaloids, Dobler et al., 2000; Hautier et al., 2008). Gas chromatography offers good separation of compounds, thus analysis by single quadrupole MS is often sufficient for targeted metabolomics, although high-resolution mass spectrometry (HRMS) using time-of-flight (TOF) instruments is increasingly used for this application as well. In contrast, LC-MS is better suited for less volatile and less thermally stable compounds. Reversed-phase (RP) liquid chromatography is suitable for separation of compounds of middle to low polarity and is therefore the most relevant technology to study PSMs, while more polar compounds such as sugars and amino acids can be analyzed using hydrophilic exchange chromatography (HILIC). Mass spectrometry can again be performed by single quadrupole MS if samples contain a limited number of target compounds with obvious (>1 Da) mass differences among themselves and with other compounds present in the sample. However, due to lower separation power and more prominent background noise inherent to liquid chromatography, targeted metabolomics applications for LC-MS more commonly use either triple quadrupole (Saremba et al., 2018), or HRMS systems, in particular quadrupole time-of-flight (QTOF) instruments. For example, the detection of sequestered benzoxazinoids in larvae of the western corn rootworm used both HRMS and single quadrupole MS successfully (Robert et al., 2017). However, the large amount of data collected by HRMS instruments allows not only for the quantification of target compounds, but has the additional benefit that if further compounds of interest were to be identified at a later stage through other approaches, their presence in a MS dataset could easily be determined without repeating the analysis.

Optimizing the extraction of target compounds may enable the reduction of background noise and compounds that could otherwise interfere with detection or data analysis. Most common solvents for PSM analysis are polar organic solvents that range from different dilutions of methanol (Robert et al., 2017; Friedrichs et al., 2022), acetonitrile (Krempl et al., 2016b), or ethanol (Sorensen et al., 2004). The chemical properties of the target compound will determine the optimal solvent necessary for extraction, with a weak solvent not extracting all target compounds, while a strong solvent may extract too many background compounds, or negatively affect chromatographic performance. For example, if exudates are to be extracted from the surface of an insect, deionized water will often dissolve all desired compounds, whereas methanol will often dissolve cuticular compounds or the content of epithelial cells. In many cases, purification of sample extracts may not be necessary, provided the extracts are diluted sufficiently before analysis (“dilute and shoot”). However, as insect samples can contain high amounts of lipids or proteins that might precipitate during LC-MS analysis, a purification step (QuEChERS, solid-phase extraction) may sometimes be necessary to prevent instrument fouling (Kanu, 2021).

For quantification and normalization of target compounds, internal standards and/or external calibration curves are essential to account for shifts in MS signal over time. Internal standards are usually added to a sample before the extraction, after tissue homogenization, thus also allowing to account for possible losses during extraction. Compounds used as internal standards could be an isotope-labeled target PSM to allow for differentiation from the native PSM (Stokvis et al., 2005), or a chemically similar compound that is not present in the sample [*e.g.*, borneol as internal standard of alpha-pinene (Sorensen et al., 2004), 4-hydroxybenzyl benzyl glucosinolate, allyl glucosinolate, as internal standard of other glucosinolates (Martin and Müller, 2007; Abdalsamee et al., 2014; Beran et al., 2018; Sporer et al., 2021), heliotrine as internal standard of senecionine alkaloids (Narberhaus et al., 2004)]. When selecting an internal standard, it is necessary to confirm that the ionization and detection of this compound is comparable with that of the target compounds. If no internal standard is available, frequent injection of reference samples within the LC-MS sample queue is important to account for MS signal shifts in that way. For quantification, the peak areas of target compounds can be compared to the internal standard peak area or those of an external calibration curve. Note that absolute quantification of MS signals is only possible for compounds for which a reference standard is available.

Given the large number of choices at all stages of designing targeted (and untargeted) metabolomic experiments, detailed and accurate reporting of sample preparation, extraction, and analysis is crucial to ensure repeatability of results, and minimum reporting standards for metabolomics experiments should be used (Sumner et al., 2007).

### Untargeted metabolomics

Detoxification and sequestration of PSMs by insects commonly involve diverse, and often unpredictable, metabolic modifications and conversions of the plant compounds by insect enzymes. An untargeted metabolomics approach can therefore serve as an important first step to determine the metabolic fate of PSMs after insect consumption. For example, specialist herbivores of Brassicaceae plants have developed many unique strategies that allow them to cope with the toxic glucosinolate PSMs of their host plant, including conjugation of the compounds with glutathione or various amino acids, desulfation of the compound, or nitrile formation upon compound breakdown (Friedrichs et al., 2022). Novel pathways of glucosinolate metabolization with different products continue to be discovered (Beran et al., 2018; Friedrichs et al., 2020), and untargeted metabolomics has been an essential tool in elucidating the fate of many PSMs (*e.g.*, Beran et al., 2018; Agrawal et al., 2021; Singh et al., 2022).

In essence, untargeted metabolomics compares two or more sample types that were exposed to different treatments and aims to identify compounds whose abundance correlates significantly with these treatments. As the insect-derived metabolites of PSMs are not always known and sometimes difficult to predict, untargeted metabolomic methods aim to capture the full set of polar and semi-polar metabolites of low molecular weight (typically between 50 and 1,000 Da) in a biological sample (Fiehn, 2002). Whereas extraction and analysis parameters can be optimized for a specific set of compounds in targeted metabolomics, the goal of untargeted metabolomics is thus to introduce as little bias to sample preparation and analysis as possible. Untargeted metabolomics commonly relies on high-resolution QTOF or Orbitrap mass spectrometry systems, as only these instruments provide sufficient temporal resolution and mass accuracy to accurately record the potentially thousands of compound ions (or ‘features’) that are commonly present in a biological sample. It is important to note here that even though untargeted metabolomics aims to provide an unbiased and complete selection of mass features (and therefore compounds), no single analytical method can fully capture the true metabolic diversity of a biological organism, and thus different metabolomics methods may have to be combined for an accurate representation of the full chemical diversity.

Data analysis is the key aspect that distinguishes untargeted from targeted metabolomics. Whereas targeted metabolomics focuses on a small number of known compounds, a single untargeted metabolomics experiment will typically generate >10,000 mass features, thus effective methods for data normalization, deconvolution (grouping of mass features belonging to the same compound), filtering, and identification of compounds of interests are essential (Lamichhane et al., 2018). To identify only those mass features associated with PSM metabolites in insects, optimal experimental design is of central importance. Ideally, the only difference between two samples in a comparison should be the presence of a PSM of interest, for example by addition of PSMs to artificial diets, or by use of mutant plants with knockouts in PSM synthesis (See Section 2). Each treatment should also be well replicated (>5 replicates per treatment) to reliably detect mass features that consistently differ between treatments. Such analyses will generally yield a subset of tens to hundreds of mass features that are differentially accumulating between treatment groups. In order to confirm these differentially expressed mass features as insect-derived metabolites of PSM, structural identification is commonly performed as the final step of untargeted metabolomic analyses.

Reliable identification of metabolites involves several different methods and approaches that differ based on instrumentation involved (Peake, 2018). GC-MS analyses generate highly reproducible molecular fragmentation patterns. Identification of compounds can thus often be achieved by comparing mass fragmentation patterns of unknown compounds to large established databases (*e.g.*, NIST/EPA/NIH). In contrast, ionization and fragmentation patterns of LC-MS are much more instrument-specific, requiring additional steps for compound identification. First, the exact mass of a compound as determined by HRMS can provide one or several possible molecular formulae. As the structure of the precursor PSM is generally known, a putative structure of a metabolite may sometimes be inferred from this formula alone. In addition, fragmentation of the unknown compound by tandem mass spectrometry (MS/MS or MS2) can provide further confirmation on a putative structure. Comparison of exact masses and molecular fragmentation patterns to public databases can sometimes result in successful identification (*e.g.*, Kyoto Encyclopedia of Genes and Genomes (KEGG) (Kanehisa and Goto, 2000), METLIN, ChemSpider, see Section 3.4), although PSMs are still underrepresented in many of these databases. If a putative compound is identified, direct comparison to an authentic standard (commercial or synthesized) is generally considered sufficient confirmation. If no putative identifications can be found or if standards are unavailable, characterization by other techniques is possible (including elemental analysis, IR, NMR, refractive index, melting point, boiling point, circular dichroism, alpha D measurement, derivatization, x-ray diffraction), although all of these generally require purification of substantial amounts of the unknown compound from its biological source. Characterization of a compound by multiple techniques is often complementary and can improve identification certainty. Among these, NMR is generally considered the key technique for unambiguous identification, but this method does require relatively large amounts of purified compound. A promising approach to facilitate rapid NMR-based identification of PSMs and their metabolites is the use of time slice LC-SPE-NMR/MS (Khakimov et al., 2016), which integrates separation and detection of compounds by LC-MS, accumulation of compound fractions on solid phase extraction (SPE) cartridges, and analysis of pooled compound fractions by NMR in a single instrument workflow.

### Labeled compounds

The identification of PSM metabolization products can be achieved through the use of labeled PSMs. One or several atoms of a PSM of interest can be replaced by stable isotopes (*e.g.*, ^13^C, ^15^N, ^2^H (deuterium), or ^17^O) or unstable radioisotopes (*e.g.*, ^14^C, ^11^C). As these labeled PSMs are then metabolized by insects, identification of conversion products is facilitated by the presence of the labeled atoms. In the field of plant herbivore interactions, several studies with labeled PSMs have been done, for example with glucosinolates, alkaloids, monoterpenoids, and cyanogenic glucosides (Brückmann et al., 2000; Chen and Halkier, 2000; Hartmann et al., 2001, 2003; Pasteels et al., 2003; Vergara et al., 2006; Kunert et al., 2008; Opitz et al., 2011; Ferrieri et al., 2013; Robert et al., 2014; Zagrobelny et al., 2014). Following the fate of labeled PSMs has enabled the characterization of their metabolization pathways and localization of metabolites in different tissues of the body (*e.g.*, Narberhaus et al., 2004). Additionally, comparing the metabolomic response of an insect after feeding on labeled and non-labeled PSMs is crucial to disentangle between PSM-induced responses and PSM metabolization.

For numerous PSMs, pure labeled versions are commercially available, and others can be synthesized from chemical building blocks. Alternatively, labeled PSMs can be produced by growing plants under a labelled CO_2_ atmosphere or with labeled N fertilization (offered as a service by some commercial providers), followed by purification of the PSMs of interest. Finally, labeled PSMs can even be produced from detached leaves or root cell cultures by feeding them labeled precursor molecules prior to stimulation of PSM production (*i.e.*, defense induction by jasmonic acid), followed again by purification of the labeled PSMs (Chen and Halkier, 2000; Hartmann et al., 2001; Pasteels et al., 2003; Kunert et al., 2008). Note that different methodological approaches result in PSMs with different extents of labeling. For PSM metabolization studies, it is therefore essential to determine which atoms in a PSM molecule are labeled (*i.e.*, by Nuclear Magnetic Resonance (NMR)), as not all resulting metabolites/fragments of a partially labeled molecule will carry the label (Narberhaus et al., 2004).

### Molecular networking analysis

Since its introduction in 2012 (Watrous et al., 2012), molecular networking provided by the Global Natural Product Social Molecular Networking (GNPS) analysis infrastructure positively impacted the chemical and biological interpretation of untargeted metabolomic analysis (Wang et al., 2016; Nothias et al., 2020). This bioinformatic tool is designed to provide as much chemical insight as possible for untargeted LC-MS2 experiments in respect to the underlying biological question. The intrinsic idea of molecular networking utilizes spectral similarity to group metabolites with the implicit assumption that similar molecular structures will generate similar MS2 fragmentation spectra. In the first step, spectral similarities are calculated between all MS2 spectra. For this process, every MS2 spectrum is displayed as a vector in a multidimensional space with each dimension correlating to a specific mass-to-charge ratio (*m/z*) with its ion intensity. Next, the angle between two vectors is calculated, resulting in a cosine-score to specify and express the similarity between two MS2 spectra. Subsequently, the matrix of spectral similarities is visually organized as a molecular networking graph with each node representing a MS2 spectrum, and edges between the nodes displaying spectral similarity above the user-defined similarity score threshold. In the last step, spectral library annotations can be propagated throughout the generated molecular networks, thereby identifying molecular families (chemical compounds of the same chemical class and/or with similar biological function and origin) and facilitating the process of structural elucidation for unknown chemical compounds. Therefore, molecular networking allows for comprehensive metabolomic characterization to provide unprecedent insight into complex insect-plant interactions, such as detecting pathway- and PSM-specific natural variation in different native plant populations (Li et al., 2015) or to reveal insect-species-specific defensive metabolites (Li, 2020).

## Molecular mechanisms underlying PSM metabolization and sequestration

### Identification of candidate genes

The analysis of the PSM fate in insects enables predictions regarding the biochemical processes involved in PSM metabolization and sequestration. The corresponding genetic pathways can be identified using transcriptome and genome assemblies, which are derived from short and long read sequencing data of insect species or populations that differs in the trait of interest. The principles, advantages and disadvantages of different sequencing technologies have been reviewed elsewhere (Rhoads and Au, 2015; Slatko et al., 2018; Hu et al., 2021). Single cell sequencing is increasingly used in other fields (Rich-Griffin et al., 2020; Dai et al., 2022) but has, to our knowledge, not yet been utilized in studies of PSM metabolism and sequestration mechanisms in insects. Moreover, the number of publicly available insect genomes and transcriptomes deposited in databases such as InsectBase2.0 (Mei et al., 2022), EnsemblMetazoa, or National Center for Biotechnology Information (NCBI) has grown enormously over the past years, providing a valuable resource for data mining and functional studies. Even if the predicted gene set is of high quality, it often remains challenging to find candidate genes, either because several different gene families could be involved and/or because the candidate gene family is large. Indeed, most classical detoxification and transporter genes, *e.g.*, cytochrome P450 enzymes, glutathione-S-transferases (GSTs), UDP-glucosyltransferases (UGTs), and ATP-binding cassette (ABC) transporters, belong to multigene families with more than 100 members in some insects (Li et al., 2007; Ahn et al., 2019; Breeschoten et al., 2021). Here, we outline approaches that can be used to identify candidates and validate them using biochemical and reverse genetic methods, and provide specific examples in Table 1.

**TABLE 1:** Examples of identified PSM metabolization and transport candidates.

Protein family	Insect species	Predicted localization	PSM	Identification of candidates	Expression system	Protein extraction		Assay substrates	Detection method	References
UDP-glucosyltransferase	*Helicoverpa armigera*	ER membrane	Gossypol	Gene expression profiling	Transient expression in Sf9 cells		Crude microsomal fraction	1-naphtol, gossypol	LC-MS/MS (product)	Krempl et al. (2016b)
UDP-glucosyltransferase	*Spodoptera frugiperda*	ER membrane	Benzoxazinoids	Sequence homology	Stable expression in High Five cells		Cell homogenate	DIMBOA, MBOA	LC-MS/MS (product)	Israni et al. (2020)
Glutathione-S-transferase	*Scaptomyza flava*	Cytosol	Isothiocyanates	Sequence homology, phylogenetic analysis	*Escherichia coli*		Affinity chromatography	1-chloro-2,4-dinitrobenzene, different isothiocanates	Spectrophotometry (product)	Gloss et al. (2014)
Glutathione-S-transferase	*Drosophila melanogaster*	Cytosol	Isothiocyanates	Gene expression profiling	*Escherichia coli*		Affinity chromatography	1-chloro-2,4-dinitrobenzene, different isothiocanates	Spectrophotometry (product)	Gonzalez et al. (2018)
Cytochrome P450 monooxygenase	*Helicoverpa armigera*	ER membrane	Gossypol	Gene expression profiling	Stable expression in Ha2302 insect cells		Microsomes	7-ethoxyresorufin and other general substrates, gossypol, nicotine	Fluorescence spectroscopy, UPLC-HRMS	Krempl et al. (2016a)
Cytochrome P450 monooxygenase	*Helicoverpa armigera*	ER membrane	Xanthotoxin, 2-tridecanone	CRISPR-Cas9 mediated knock-out and perfomance assays	Baculovirus-mediated expression in High Five cells		Microsomes	Xanthotoxin, 2-tridecanone	UPLC-MS/MS (substrate)	Wang et al. (2018)
Flavin-dependent monooxygenase	*Tyria jacobaeae*	Extracellular	Pyrrolizidine alkaloids	Inhibitor experiments, protein purification from larval hemolymph	*Escherichia coli*		Solubilization of inclusion bodies and refolding	different pyrrolizidine alkaloids	Spectrophotometry (NADPH decrease)	Lindigkeit et al., 1997; Naumann et al., 2002
Glucoside hydrolase family 13	*Bemisia tabaci*	Extracellular	Glucosinolates	Sequence homology, phylogenetic analysis	Stable expression in S2 cells		Crude culture medium	different glucosinolates	LC-MS/MS (product)	Malka et al. (2018)
Phenolic glucoside malonyltransferase	*Bemisia tabaci*	Cytosol	Phenolic glycosides	KEGG pathway analysis of predicted genes, plant-mediated RNAi	Baculovirus-mediated expression in Sf9 cells		Affinity chromatography	different phenolic glycosides	UPLC-QTOF/MS	Xia et al. (2021)
Arylsulfatase	*Plutella xylostella*	Extracellular	Glucosinolates	Protein purification from larval gut protein extracts	Transient expression in Sf9 cells		Crude culture medium	4-methylumbelliferyl sulfate, different glucosinolates	HPLC-UV (product)	Ratzka et al. (2002)
Arylsulfatase	*Psylliodes chrysocephala*	Cell embrane	Glucosinolates	Sequence homology, phylogenetic analysis	Transient expression in Sf9 cells without TMD		Crude culture medium	4-nitrocatechol sulfate, different glucosinolates	LC-MS/MS (product)	Ahn et al. (2019)
Arylsulfatase	*Bemisia tabaci*	Extracellular	Glucosinolates	Sequence homology, gene expression profiling	Transient expression in Sf9 cells		Affinity chromatography	different glucosinolates	LC-MS/MS (product)	Manivannan et al. (2021)
Nitrile Specifier Protein	*Pieris rapae*	Extracellular	Glucosinolates	Protein purification from larval gut protein extracts	*Escherichia coli*		Crude *E. coli* extracts	Benzyl glucosinolate	GC-MS, GC-FID	Wittstock et al. (2004)
ABC transporter	*Chrysochus auratus*	Cell membrane	Cardenolides	Sequence homology, phylogenetic analysis	Baculovirus-mediated expression in Sf9 cells		Membrane vesicles	verapamil, different cardenolides	Spectrophotometry (released phosphate)	Kowalski et al. (2020)
MFS transporter	*Phyllotreta armoraciae*	Cell membrane	Glucosinolates	Sequence homology, phylogenetic analyses	High Five cells, *Xenopus laevis* oocytes		None (intact cells, oocytes)	different glucosinolates, other non-host plant glucosides	LC-MS/MS (product)	Yang et al. (2021)

ER: endoplasmic reticulum; DIMBOA: 2,4-dihydroxy-7-methoxy-(2H)-1,4-benzoxazin-3(4H)-one; KEGG: Kyoto Encyclopedia of Genes and Genomes; MBOA: 6-methoxy-benzoxazolin-2-one.

#### Transcriptomic analyses

The identification of candidate genes in multigene families can be approached by an initial annotation of all putative members in a predicted gene set based on sequence similarity to known gene family members from other insect species, using basic local alignment search tools (BLAST). From this comprehensive list of genes, candidates can be filtered by gene expression analyses, comparing for instance overall levels of gene expression, expression in different insect tissues, or in different developmental stages. Alternatively, candidate genes are selected based on their inducibility in response to ingestion of specific PSMs compared to a control treatment without PSMs. However, genes involved in metabolization and sequestration could also be expressed constitutively, especially in specialist herbivores, which are continuously exposed to specific PSMs. The most comprehensive method to analyze gene expression is by RNA sequencing (RNA-Seq). RNA is extracted from dissected tissues or whole insects with several replicates (≥3) per treatment or tissue. Comparable amounts of RNA from each sample are used to prepare a sequencing library and sequenced using short read (*e.g.*, Illumina) or long read sequencing technologies (*e.g.*, PacBio Iso-Seq). The number of sequenced reads depends, in addition to the availability of resources, on the sequencing method, the expected transcript abundance, and the complexity of the starting sample (*e.g.*, individual tissues or whole insects). Sequenced reads are then mapped to the predicted gene set using available tools. RNA-Seq studies usually reveal hundreds of differentially regulated genes even within a single tissue, showing that insect molecular responses to PSMs are complex (*e.g.*, Robert et al., 2013; Malka et al., 2018; Li Q et al., 2019; He et al., 2022).

Some studies employed gene expression profiling as a tool to predict metabolic pathways involved in PSM metabolism (*e.g.*, Crava et al., 2016; He et al., 2022). However, studies with the cotton bollworm *Helicoverpa armigera* have shown that upregulation of detoxification genes may not be directly linked to PSM metabolism (Krempl et al., 2016a). The ingestion of gossypol, a reactive polyphenolic defense compound found in cotton plants, strongly induced expression of the cytochrome P450 monooxygenase CYP6AE14 in cotton bollworm larvae (Mao et al., 2007; Celorio-Mancera et al., 2011), and silencing *CYP6AE14* gene expression negatively influenced larval growth after gossypol feeding (Mao et al., 2007). Although these findings strongly suggested that CYP6AE14 was involved in gossypol detoxification, there was no evidence for CYP6AE14-mediated metabolism of gossypol in enzyme assays with recombinant enzymes. In addition, *CYP6AE14* expression was also induced upon ingestion of other plant toxins such as nicotine, which suggests that the upregulation of *CYP6AE14* expression constitutes a more general stress response (Krempl et al., 2016a). These findings highlight that functional studies are critical to validate the role of a candidate gene identified based on transcriptome profiling and to differentiate between general and specific transcriptional responses to PSMs.

#### Phylogenetic analyses

Candidate genes can be sought by analyzing the diversification of the target gene family across different insect species. The number of genes within a family can vary dramatically even among closely related insect species, which could be the result of adaptive processes driven by PSMs or due to random “gene birth-and-death” processes (Nei and Rooney, 2005; Demuth and Hahn, 2009; Calla et al., 2017). Species included in such a phylogenetic study should be ideally closely related to the species of interest (*e.g.*, belong to the same genus, subfamily, or family) but differ with respect to the trait under study. After gene annotation, phylogenetic analyses are performed to analyze the diversification pattern using nucleotide or amino acid sequence alignments. The resulting phylogenetic trees will reveal genes that are conserved and those that have duplicated in a lineage-specific manner, which might be linked to the emergence of an evolutionarily novel function in PSM detoxification and sequestration (*e.g.*, Ahn et al., 2019; Yang et al., 2021; Lin et al., 2022). Gene family evolution can also be analyzed using CAFE (Computational Analysis of gene Family Evolution), a statistical method that estimates gene gains and losses across a species phylogeny based on birth and death models (Bie et al., 2006; Han et al., 2013).

#### Proteomic analyses

Proteomic analyses can be a powerful tool to discover enzymes that are involved in PSM metabolization. One particular advantage of proteomic analyses is the fact that they allow to elucidate new pathways that may not be highlighted during phylogenetic and/or transcriptomic studies (*e.g.*, conserved pathways which are constitutively expressed). Two main approaches can be undertaken to identify proteins that interact with PSMs.

Activity-guided protein fractionation coupled to mass spectrometry aims to purify and identify the protein(s) responsible for PSM metabolization in a complex mixture (Issaq et al., 2002). In brief, a crude protein extract is prepared from whole insects or dissected insect tissues by homogenization in a suitable extraction buffer, and applied to a column separating the proteins based on their biophysical properties (*e.g.*, size, charge, glycosylation) using for instance fast liquid protein chromatography (FPLC). The obtained protein fractions are then screened for activity by carrying out enzyme assays (See Section 4.2). Active fractions are then combined and subjected to another chromatographic step or to protein sequencing using mass-spectrometry. For an overview of sample preparation methods for proteomic analyses refer to Rogers and Bomgarden (2016). Predicted amino acid sequences are back-translated into nucleotide sequences that are aligned with the insect’s predicted gene set. Genes matching the purified protein(s) are validated as described below. The limitation of this approach is that activity-guided protein fractionation is largely restricted to soluble proteins and requires the ability to screen numerous protein samples for activity in a short period of time. Yet, it allowed identification of a nitrile specifier protein in the gut of cabbage white butterfly larvae, a protein unrelated to other functionally characterized proteins, that enables the insect to overcome the glucosinolate-based defense of their brassicaceous host plants (Wittstock et al., 2004).

Mass spectrometry-based proteomic studies can be used to identify and characterize protein-ligand complexes. Proteomic analyses include strategies based on limited proteolysis and labeling, or on the use of protein denaturant and probing of folding/unfolding protein reactions (Kaur et al., 2018). These proteome-wide structural methods include Limited Proteolyses (LiP) (Feng et al., 2014; Liu and Fitzgerald, 2016), chemical cross-linking (XL-MS) (Tang and Bruce, 2010), Hydroxyl Radical Footprinting (HRF) (Espino et al., 2015; Chea and Jones, 2018), Drug Affinity Responsive Target Stability (DARTS) (Lomenick et al., 2009), Pulse Proteolysis (PP) (Liu et al., 2011; Chang et al., 2012; Adhikari and Fitzgerald, 2014; Zeng et al., 2017), Stability of Proteins from Rates of Oxidation (SPROX) (Dearmond et al., 2011; Tran et al., 2014), and Thermal Proteome Profiling (TPP) (Molina et al., 2013; Savitski et al., 2014). The strengths and weaknesses of each of these methods were thoroughly reviewed in Kaur et al., 2018. Yet, to date, only XL-MS, HRF, and TPP enabled the study of the interactions between a ligand (*e.g.*, PSM) and proteins *in vivo.* The proteins identified as interacting with the tested ligand should further be examined to disentangle whether the interaction is due to targeting of proteins, metabolization and/or sequestration machinery, and untargeted effects (see Section 4.2). The interaction between the identified proteins and PSMs can further be predicted using modern multi-core computational simulations to identify the docking of small molecular weight molecules to proteins (Hassan et al., 2005; Lecina et al., 2017). Recently, a novel chemogenomic algorithm using machine learning was developed to rapidly characterize the proteochemical space and to increase the rapidity and resolution of protein-ligand interactions (Li S et al., 2019). Although these techniques have been mostly applied to drug discovery and drug off-target effects on microbial pathogens, mouse and human cells, their applications to unravel detoxification and/or sequestration mechanisms in insects is promising.

Protein interaction networks can be constructed to gain further information about metabolization pathways. The Search Tool for the Retrieval of Interacting Genes/Proteins (STRING) can be used for the identification of direct physical and undirect functional correlations between proteins and to score Protein-Protein Interactions (Szklarczyk et al., 2021). The STRING database contains more than 14 000 organisms and was successfully used to discover protein networks involved in xenobiotic detoxification in insects (Zhang and Zhang, 2019b; Cao and Cheng, 2020).

#### Co-expression networks with multi-omics datasets

Gene co-expression networks identify groups of genes with similar expression patterns across tissues and treatments, with the underlying assumption that genes involved in the same biological processes, such as insect response to a particular host plant, will be co-regulated under the same environmental or developmental conditions (Wisecaver et al., 2017). In co-expression networks, genes are represented by nodes or vertices, and co-expressed genes are connected by edges, which may be weighted to indicate the strength of the co-expression association between two genes. Clusters or modules of co-expressed genes can be identified with a range of clustering algorithms, providing the researcher with groups of functionally related genes. Downstream analyses including functional enrichment or differential expression are overlaid on the clusters to identify modules relevant to a particular process or question (Delli-Ponti et al., 2020). Gene co-expression analyses have been successfully applied in a range of contexts including pathway discovery in plant specialized metabolism (Chung et al., 2020) and identification of virulence factors in plant-insect interactions (Chen et al., 2020) and could be a powerful approach for integrating multi-omics data to identify genes involved in PSM metabolization by insects.

When designing a gene co-expression analysis, it is useful to maximize variation by collecting data from a wide range of treatments and conditions. In general, inclusion of more tissue types and treatments will yield a more robust network (Wisecaver et al., 2017). In the case of detoxification of PSMs, this may include dissected insect tissues (being sure to include both tissue where detoxification is thought to occur and tissue where it is not), whole insects feeding on a range of host plants, and whole insects after feeding on artificial diet either supplemented with or lacking the PSM of interest. One may also include multiple strains of the focal insect if available. For example, when studying detoxification of benzoxazinoids in *Spodoptera frugiperda*, the fall-armyworm, inclusion of both the rice-feeding strain and the maize-feeding strain would likely increase variation in detoxification gene expression because maize produces benzoxazinoids while rice does not (Silva-Brandão et al., 2021). Quantification of gene expression commonly involves RNA sequencing with a reference-based or *de novo* assembly (Hafeez et al., 2021), but quantitative proteomics is also compatible with co-expression networking (Gibbs et al., 2013). Moreover, co-expression analyses can be conducted without collecting any new data by compiling publicly available datasets from disparate studies on the same organism (Wisecaver et al., 2017; Tian et al., 2021).

Several popular networking approaches have been developed, and the appropriate choice depends on the context and questions being asked. Targeted approaches use “bait” genes that are already known to be involved in the process of interest to search for missing pathway components. CoExpNetViz (Tzfadia et al., 2016) is a simple and easy-to-implement tool available as a package within Cytoscape (Shannon et al., 2003). With the bait gene approach, the best candidate genes for further study are those that are highly co-expressed with most or all bait genes.

Other networking tools such as Weighted Gene Co-expression Network Analysis (WGCNA, (Langfelder and Horvath, 2008)) or the mr2mods workflow (Wisecaver et al., 2017) take an all versus all approach and can generate hundreds of clusters of co-expressed genes. With no “bait” genes to identify a cluster related to PSM detoxification, other strategies including gene functional enrichment and differential expression can be superimposed on the network to identify clusters of interest (Delli-Ponti et al., 2020). For example, a co-expression networking study on whitefly host plant selection identified modules of interest by first looking for co-expression modules with higher gene expression in the whitefly salivary glands and midgut, and then performing KEGG and Gene Ontology GO; (Ashburner et al., 2000; Gene Ontology Consortium, 2021) enrichment analyses on the modules of interest. In this case, three modules with higher expression in the salivary glands or midgut and enriched for GO terms such as “peptidase activity” and KEGG pathways such as “lysosome” were identified as host selection modules. The authors noted that these modules contained potential detoxification-related P450s and UGTs but did not functionally validate these candidates (Tian et al., 2021).

Integration of co-expression networks with multi-omics datasets allows for a more complete model of a biological process and helps identify the top candidate genes for functional characterization. The simplest strategy for integration of metabolomics or microbiome data is to map these data onto an existing co-expression network generated using one of the previously discussed strategies. For example, if a PSM modification is identified in an untargeted metabolomics experiment, the correlation between the abundance of the modified metabolite and gene expression could be mapped onto the network to highlight modules and genes whose expression correlated with this modification across tissues and treatments. Other emerging tools use more sophisticated algorithms to integrate multi-omics data into a single network (Zhou et al., 2020). For example, PIUMet uses a prize-collecting Steiner forest algorithm to integrate untargeted metabolomics and proteomics datasets for identification of metabolite and protein features that are perturbed during disease (Pirhaji et al., 2016). Though designed to study human disease, PIUMet may serve as a model for the use of similar techniques in plant-insect interactions and PSM detoxification.

### Validation of candidate genes

#### Functional characterization of candidates *in vitro*

To date, there is a strong discrepancy regarding the number of studies investigating the effects of PSMs on gene expression and those validating the function of individual genes using heterologously expressed proteins (Amezian et al., 2021). The general workflow consisting of protein expression, protein harvesting and purification, and testing of activity has to be tailored towards the protein of interest. Briefly, candidate genes are PCR-amplified from cDNA or synthesized in a codon-optimized version for the selected protein expression system, and subcloned in an expression vector containing additional elements required for protein expression such as a suitable promotor sequence. Protein expression can be carried out in different systems including bacteria (*Escherichia coli*), yeast (*e.g.*, *Saccharomyces cerevisiae* or *Pichia pastoris*), and insect cells (*e.g.*, Sf9 and Sf21, derived from ovaries of the fall armyworm, *S. frugiperda*, High Five, derived from ovaries of the cabbage looper *Trichoplusia ni*, and Schneider 2 (S2) derived from *Drosophila melanogaster* embryos). *Xenopus laevis* oocytes are used for expression and biochemical characterization of membrane transporters and other membrane proteins.

The *E. coli* expression system has many advantages and is usually preferred if the protein does not require posttranslational modifications (*e.g.*, Francis and Page, 2010; Rosano and Ceccarelli, 2014). If posttranslational modifications are essential for activity, a eukaryotic expression system must be used. Modifications in insect cells are likely to be more similar to the native state than modifications in yeast. Insect cells allow for transient expression, baculovirus-mediated transient expression for high yield, and stable expression. Protocols depend on the system that is used and are provided by the manufacturers. Further steps to retrieve the recombinant protein for biochemical assays depend on its localization, *e.g.,* in the cytosol, the cell membrane, the endoplasmic reticulum, or in the expression medium as a secreted protein.

Depending on the expected activity, enzymatic assays are carried out with the recombinant enzyme, substrate(s), cofactors required for enzyme activity, and an appropriate buffer. Background controls include assays with boiled protein, without substrate, or without protein. Assay products may be analyzed by chemical analytical methods or other methods developed for specific enzyme classes (see Table 1 for examples).

In the *Xenopus* system, complementary RNA (cRNA) is synthesized and injected into oocytes using a microinjection set-up. As negative control, oocytes are injected for example with water. A detailed protocol for these steps is provided in (Jørgensen et al., 2016). Assays are performed directly with the transporter-expressing and control oocytes, *e.g.*, by adding the substrate(s) into the buffer to detect import activity, or by injecting the substrate into the oocyte to detect export activity (*e.g.*, Strauss et al., 2013; Jørgensen et al., 2017).

#### Functional characterization of candidates *in vivo* by *RNAi*

RNA interference (RNAi), a conserved post transcriptional gene silencing mechanism, represents the most commonly used method for studying the function of genes in insects. In RNAi experiments, the nucleotide sequence of the candidate gene is used as a template to synthesize double stranded RNA (dsRNA), which is injected into the insect body or administered orally, for example via an artificial diet or using plants expressing dsRNAs. The exogenous dsRNA is taken up into cells primarily by endocytosis and processed into short (19–21 base pairs) interfering RNAs (siRNAs) that trigger the siRNA pathway, resulting in the degradation of the target messenger RNA (mRNA) through sequence complementarity (Zhu and Palli, 2020). As negative control, insects are treated with another dsRNA that should ideally have no complementary mRNA in the insect and thus have no effect on gene expression. dsRNAs targeting the green fluorescent protein (GFP) and synthetic scrambled dsRNA with the same nucleotide composition as the gene-specific dsRNA but a different sequence are frequently used as controls. A common challenge in RNAi experiments is the avoidance of off-target effects, *i.e.,* the simultaneous knock-down of additional non-target genes. If the treatment reduces expression of several genes, it may not be possible to assign the phenotype to one specific gene. Open-source software such as siRNA-Finder (si-Fi) can help to design dsRNAs for specific gene knock-down (Lück et al., 2019).

The impact of the dsRNA treatment on gene expression is evaluated by quantitative reverse transcription PCR (qRT-PCR) experiments, comparing the transcript abundance of the candidate gene normalized to a reference gene between the different treatments. To determine the specificity of RNAi, the expression level of genes with high sequence similarity to the target gene should be analyzed as well. Important considerations for the design of qRT-PCR studies are summarized in the MIQE guidelines (minimum information for publication of quantitative real-time PCR experiments) (Bustin et al., 2009; Taylor et al., 2010). Finally, the resulting phenotype of reduced gene expression is analyzed, for example by measuring the fate of PSMs or the level of enzyme activity in insects.

The susceptibility towards RNAi and its efficiency varies considerably among insects (Terenius et al., 2011; Zhu and Palli, 2020; Silver et al., 2021); thus, RNAi experiments can require extensive optimization, *e.g.*, regarding the amount and length of the dsRNA, the dsRNA delivery mechanism, and time point of application (Mehlhorn et al., 2021). More efficient techniques to deliver dsRNA and trigger an RNAi response are being developed in particular for RNAi-mediated pest control, which will also facilitate characterization of gene function in insects that less susceptible to RNAi with current methods (Silver et al., 2021).

#### Functional characterization of candidates *in vivo* by *CRISPR-Cas9*

The CRISPR-Cas9 system is another valuable tool to study gene function through genome editing (Jinek et al., 2012). The Cas9 enzyme is an RNA-guided endonuclease that introduces double-strand breaks in target DNA, which are repaired by non-homologous end joining. This repair mechanism frequently introduces small insertions or deletions that can render a target gene non-functional. The specific binding of Cas9 to the target DNA sequence is mediated by a guide RNA (gRNA) consisting of a target gene-specific CRISPR RNA (crRNA) and the *trans*-activating CRISPR RNA (tracrRNA) that interacts with the Cas9 nuclease. The Cas9-gRNA complex is also referred to as ribonucleoprotein (RNP). DNA recognition and cleavage by Cas9 requires the presence of the protospacer adjacent motif (PAM) sequence of NGG nucleotides directly downstream of the target sequence. Several web-based tools are available for crRNA design and off-target prediction (Cui et al., 2018). It is generally recommended to design several crRNAs and test their efficiency in guiding Cas9-mediated cleavage *in vitro*. The custom-designed crRNA, tracrRNA, and recombinant Cas9 can be ordered from different companies, but these components can be also prepared in the laboratory if necessary.

The basic workflow to obtain genome-edited insect lines comprises 1) microinjection of the RNP into freshly laid eggs, 2) screening of adults for target mutations (G_0_), 3) crosses of genome-edited adults and screening for transmission of target mutations to G_1_, 4) set-up of mutant lines. The timing and method of egg injection are critical parameters that determine the efficiency of genome editing. Recent publications by Kotwica-Rolinska et al. (2019) and Tang et al. (2022) provide detailed workflows for the set-up and optimization of CRISPR-Cas9 experiments in a hemipteran and a lepidopteran non-model insect, respectively. These methods preclude viviparous insect species and species whose eggs are not easily accessible for injection. The recent development of “direct parental” CRISPR (DIPA-CRISPR) now also enables genome-editing of insects in which embryo injection is not feasible (Shirai et al., 2022). DIPA-CRISPR relies on the injection of females undergoing vitellogenesis with RNP, which appears to be non-selectively incorporated into the developing oocytes. This method resulted in gene editing efficiencies of up to 21.8% in the German cockroach, *Blattella germanica*, and over 50% in the red flour beetle, *Tribolium castaneum* (Shirai et al., 2022).

Depending on the generation time of the target insect, it can take 1 year or even longer to obtain stable mutant lines. This time investment can be worthwhile as it allows a comprehensive analysis of costs and benefits of PSM metabolization and sequestration.

In the cotton bollworm, *H. armigera*, CRISPR-Cas9 was used to knock-out an 85 kb genomic fragment carrying a cluster of nine cytochrome P450 genes by using gRNAs targeting the genes at the beginning and the end of the cluster (Wang et al., 2018). The knock-out line was more susceptible to several PSMs and insecticides than the corresponding wild type, and functional studies with recombinant enzymes revealed distinct P450 enzymes to be responsible for metabolism of specific PSMs and an insecticide. This study demonstrates that CRISPR-Cas9 can be used for larger scale screens aimed to understand the function of genes that have recently diversified through tandem-duplication events.

## PSM localization

The distribution of PSMs and metabolization products within an herbivore body can be crucial to identifying the molecular and biochemical mechanisms of detoxification and sequestration, but also to tackling their possible effects on the insect biology. Localizing PSMs can be achieved through the metabolomic analysis of individual tissues, mass spectrometry imaging, immunohistochemistry, *in situ* hybridization, or even positron emission tomography (PET) imaging.

### Organs and organoids

PSM localization can be assessed in single organs after dissection of the insect specimens (See Section 2.3). Yet, organs can also be grown *in vitro.* Spheroids and organoids have been developed for drug discovery and personalized medicine to encapsulate the complexity and heterogeneity of responses to drugs in complex genetic and molecular environments (Abbott, 2003; Kamb, 2005; Pampaloni et al., 2007). Spheroids and organoids are three-dimensional (3D) cell cultures that mimic the cellular architecture and behavior of animal tissues by allowing cell-cell and cell-extracellular matrix interactions (Baker and Chen, 2012; Tanner and Gottesman, 2015; Zanoni et al., 2020). Spheroids are a compact and spherical aggregation of cells grown from different tissue-specific stem cells or regenerating cells of an organism (Yamada and Cukierman, 2007; Hirschhaeuser et al., 2010; Weiswald et al., 2015). Microfluidic techniques for spheroids involve emulsion, microwells, U-shaped microstructures, or digital microfluidics. The specific designs, strengths, and weaknesses of these techniques are reviewed in Moshksayan et al., 2018, Białkowska et al., 2020, and Gunti et al., 2021. Organoids are 3D cell cultures grown from stem cells that differentiate in a multistep procedure (Gunti et al., 2021). Achieving the full differentiation of an organoid requires the balanced addition of chemicals involved in the organ development (*e.g.*, growth factors). Given the limited knowledge available of insect organogenesis mechanisms, preparing insect-derived organoids may be still challenging. Yet, developing spheroids and/or organoids from insect cells would be a corner stone in advancing the fields of plant-herbivore interactions and pest management (Swevers et al., 2021).

### Mass spectrometry imaging

Directly mapping PSMs in a histological section allows distinction of the exact tissue and cells in which they are present. However, not all compounds can be detected by standard histological procedures (for a review on strategies for annotation and identification of small molecules see Baquer et al., 2022). Mass spectrometry imaging (MSI) provides simultaneous and spatially resolved analysis of molecular species in an untargeted fashion, and therefore, has been widely used to map the distribution of a broad variety of molecular compounds in tissues, organs, or whole organisms. For instance, MSI enabled drug-pathway analysis in pharmaceutical research and provided spatial lipidomic and proteomic characterization for several invertebrates (Niehoff et al., 2014; Khalil et al., 2015; Yang et al., 2020).

Among MSI methods, matrix-assisted laser desorption/ionization (MALDI) MSI represents one of the most advanced techniques for bioanalytical research. In the first step, the biological sample, usually a histological cross-section of organs or whole-body section, is carefully embedded in a matrix of UV-absorbing low molecular weight molecules that extracts and crystalizes the analytes of interest. In this context, the MALDI matrix has to be carefully selected depending on the analytes of interest (Calvano et al., 2018; Zhou et al., 2021). Next, the section is scanned with a defined step-size (x- and y- grid) and for every measurement event, the analyte-matrix cocrystals are irradiated by laser pulses resulting in rapid heating, localized ablation and subsequent ionization of the analytes of interest. The ionized analytes are then transferred into the mass spectrometer by an electrostatic field and analyzed in the mass analyzer to determine their *m/z*. A mass spectrum, consisting of individual *m/z* values with their corresponding intensities, is obtained for every measurement event. Using dedicated software tools, the chemical information is combined with the spatial information (based on the x,y-coordinates of the grid) into a heat map image displaying the relative distribution of the selected *m/z* values for the analyzed region of interest (Spengler, 2015). Recent technological advances for MALDI MSI enabled high-resolution biomolecular imaging for a plethora of different sample types (*e.g.*, mammalian- and plant tissues) (Klein et al., 2015; Crecelius et al., 2017; Kompauer et al., 2017; Hohenstein et al., 2019; Villete et al., 2020).

However, despite its great potential, only a few studies have utilized MSI to study the fate of plant secondary metabolites in insects. For instance, the spatiotemporal distribution of glucosinolates in hymenopteran larvae in whole body cross-sections was analyzed for different time points (Abdalsamee et al., 2014). The authors were able to visualize the absorption of glucosinolates in the front part of the gut to avoid the activation of these secondary metabolites in the gut. By combining high resolution in mass and space, MALDI MSI was used to reveal differences and provide spatially resolved molecular insight for plant toxin sequestration in the monarch butterfly (*D. plexippus*) and the common crow (*Euploea core*) at the low-micrometer scale (Dreisbach et al., 2022). Therefore, both studies demonstrate the potential of MSI to investigate the biochemistry of insect-plant interactions in the spatial context of insect tissues and cells.

Alternatively, Desorption Electrospray ionization (DESI) and Laser Ablation Electro Spray ionization (LAESI) are ambient ionization techniques for MSI, which require minimal to no sample preparation at all. DESI utilizes ionization principles of electrospray (ESI) by pneumatically directing an electrically charged mist to the sample surface. Subsequently, analytes of interest are desorbed via splashed droplets, ionized and transported into the mass spectrometer for determining their *m/z* ratio. In contrast, LAESI utilizes mid-infrared (IR) laser pulses to ablate material from the sample surface. Next, an electrospray mist is pneumatically directed towards the ablation cloud causing ionization of the analytes of interest, which are then transmitted into the mass spectrometer. Both methods have been applied in pharmaceutical and natural products research, especially when mapping small lipophilic molecules, including plant metabolites in roots and alkaloids in whole body sections of alkaloid-sequestering poison frogs (Kulkarni et al., 2018; Jeckel et al., 2020). However, the spatial resolution of these methods is far lower than when compared to MALDI. Whereas typical DESI- and LAESI MSI experiments are conducted with 50–100 µm spatial resolution, recent developments for commercial MALDI MSI systems provide for up to 5 µm spatial resolution - thereby approaching the (sub)-cellular level. In conclusion, the ionization technique has to be selected in respect to the experimental design and underlying biological question (*e.g.*, analytes of interest, sample type and conditions).

### Immunochemistry

Producing antibodies specifically targeting small molecules, such as most PSMs, requires conjugating them to an immune response elicitor, such as a protein (*i.e.*, hapten-carrier conjugate). The production of a hapten-carrier conjugate might not be completely specific, because it can elicit non-specific immune response due to the protein carrier, but production of antibodies targeting PSM has been done and it could be an interesting method to have available (Pongkitwitoon et al., 2010; Sakamoto et al., 2018). One way to use immunohistochemistry to localize the detoxification and sequestration in the histological section is to use the enzymes and proteins responsible for these processes (Jones et al., 2001; Daimon et al., 2008). Jones et al. (2001) were able to purify an antibody specific for myrosinase enzymes of cabbage aphid *Brevicoryne brassicae* that does not cross react with the plant myrosinase. This antibody allowed mapping the spatial organization of glucosinolate-myrosinase system in the aphid and comparing it to the plant organization (Bridges et al., 2002). The effects of PSMs on non-specialized insects can also be investigated by targeting digestive enzymes in specific parts of the gut (Mashhoor et al., 2021).

### *In situ* hybridization

Fluorescent *in situ* hybridization (FISH) of RNA provides information on the spatial distribution of gene transcripts in their native cellular environment. The most commonly used RNA probes are indirect immunochemical methods, where the probe is labeled with a reporter molecule that will be bound to antibodies that, in turn, are conjugated to alkaline phosphatase, peroxidase, fluorescein, rhodamine, or colloidal gold. Commercially pre-labeled probes allow investigating the distribution of multiple transcripts by using different fluorophores that emit specific detectable fluorescent signals (Kwon, 2013). The identification of transcript-expressing cells can help understanding where and how detoxification and sequestration processes are occurring in the herbivore body (Kliot et al., 2014; Sun et al., 2019). For example, the labeling of three transcripts of glucosinolate sulfatase indicated that they are inducible and co-expressed in the same cells of the internal layer of the midgut of diamondback moth (*Plutella xylostella*) (Sun et al., 2019).

FISH has also been used to detect and investigate the functions and distribution of symbiotic bacteria in insects (for more details, see Kliot et al., 2016). By targeting intracellular components, FISH allows identification and investigation of obligatory intracellular symbionts that would not be possible to isolate or cultivate in artificial media (Moss et al., 2018). As the role of the microbiome in the detoxification of PSM has been widely recognized lately, mapping the distribution of these organisms in herbivores could be an insightful instrument to be used with other identification methods.

### Positron emission tomography imaging

Positron Emission Tomography (PET) is a promising avenue for the study of PSM distribution in herbivores. PET measures *in vivo* concentration and distribution of radiolabeled compounds in a non-invasive manner. With the development of PET scanners with high resolution (1 vs. 4 mm), it became possible to use PET in research with small animals and plants. The main advantage is that PET imaging is non-invasive and does not require animal sacrifice (Hutchins et al., 2008). For example, PET imaging allows to investigate the transport of nutrients, phytohormones and photoassimilates in plants [for a review on the use of PET in plant studies, see Mincke et al. (2021)] and could be further used for feeding herbivores.

## Conclusion and perspectives

This review emphasizes cutting-edge techniques, from the field and beyond, that empower researchers to study herbivore resistance strategies to PSMs. These cutting-edge methods enable scientists to characterize the processes that underlie PSM metabolization, diversion (incl. sequestration), or rapid elimination of metabolized products. The unprecedented technological progresses allow researchers to increase the resolution of our understanding of the processes from whole organisms to single cells. While tolerance strategies were not under the scope of this work, several techniques described presently can be used to assess insensitivity, exclusion, and direct excretion. Further microbiological studies can be conducted to assess the contribution of associated organisms (microbes from the digestive system) to PSM resistance in insects (Adams et al., 2013; Boone et al., 2013; Hammerbacher et al., 2013; Mason et al., 2014; Vilanova et al., 2016; Welte et al., 2016; Jing et al., 2020; Shukla and Beran, 2020). Finally, field trials and experimental selection assays can further elucidate the ecological and evolutive significance of PSM resistance in insects (Erb and Robert, 2016; Zhang et al., 2019a; Machado et al., 2020; Petschenka et al., 2022).
